# Floristic diversity and species composition along altitudinal gradient in the alpine ecosystem of the cold desert region in Western Himalaya, India

**DOI:** 10.3389/fpls.2024.1469579

**Published:** 2024-11-27

**Authors:** Amit Bahukhandi, K. Chandra Sekar, Vikram S. Negi, Kapil Bisht, Deep C. Tiwari, Poonam Mehta, Shashi Upadhyay, Sazada Siddiqui, Amel Ayari-Akkari

**Affiliations:** ^1^ G.B. Pant National Institute of Himalayan Environment (GBPNIHE), Almora, Uttarakhand, India; ^2^ Garhwal Regional Centre, GBPNIHE, Srinagar Garhwal, Uttarakhand, India; ^3^ Department of Botany & Microbiology, Hemvati Nandan Bahuguna Garhwal University (A Central University), Pauri Garhwal, Uttarakhand, India; ^4^ Department of Botany, Hermann Gmeiner Degree College, Bhimtal, Uttarakhand, India; ^5^ Department of Biology, College of Science, King Khalid University, Abha, Saudi Arabia

**Keywords:** Trans-Himalaya, alpine region, Nelang Valley, floristic diversity, high altitude vegetation, biodiversity conservation

## Abstract

In India, the Trans-Himalayan zone lies in the rain shadow of the main Himalayan region and is usually described as a “high-altitude cold desert”. These regions are represented by sparse but unique vegetation composition. The present study is an attempt to investigate the vegetation composition in the alpine ecosystem of the cold desert landscape of the Nelang Valley in Western Himalayas (Uttarakhand), India. The result of the study reveals the existence of a total of 68 taxa of higher plants (56 genera and 28 families). These include herbs (51 species), shrubs (13 species), climbers (one species), and trees (three species). Herbaceous species (52 species) contributed the maximum species richness; Artemisia, Astragalus, and Juniperus were the dominant genera, followed by Aster, Lonicera, Oxytropis, Poa, and Salix. The valley showed irregular distribution of plant species richness along the altitudinal gradient, and maximum taxa exhibited between 3,500 m and 3,600 m of altitude band. In the Nelang Valley, the total number of phytodiversity individuals exhibited a significant linear decline with increasing altitude (3,100–4,300 m). Further, the study recorded 33 important medicinal plants used in the traditional system of herbal medicine in the Himalayan region. Juniperus semiglobosa shows dominance with a maximum important value index (IVI) (31.77), followed by Pinus wallichiana (29.19) and Cedrus deodara (10.72) in the treeline ecotone of the valley. Rapid Threat Assessment shows that *Artemisia dubia* and *Artemisia roxburghiana* were the most vulnerable herb species in the region. The information thus generated will be useful for suggesting ecological management and conservation planning.

## 1 Introduction

Mountains are remarkably diverse and globally important as centers of biological diversity; they are home to 50% of global biodiversity hotspots and harbor nearly a quarter of the world’s forests (Spehn and Körner, 2005; Spehn et al., 2010; Wani et al., 2022a). The alpine zone is globally distributed, from polar to tropical latitudes, and covers 3% of the global terrestrial land area (Körner, 1995). The overall global vascular plant species richness of the alpine life zone was estimated to be approximately 10,000 species, 4% of the global number of higher plant species (Spehn and Körner, 2005). Among various mountain ecosystems in the world, the Himalayas holds a special significance for being the youngest, most dynamic, and most diverse ecosystem, and it represents the Himalayan Biodiversity Hotspot (Rawal et al., 2013). Plant communities and species diversity often show distinctive responses to elevation gradient, and therefore, the mountain region is the storehouse of global biodiversity (Körner, 2000, 2007; Spehn et al., 2010). Globally, the alpine zone covers approximately 2.6% of the terrestrial landform (Körner et al., 2023), while in the Himalayan region, it covers nearly 33% (26% of the area is vegetated, and 7% area falls under perpetual snow or barren rocks) of the geographical area (Lal et al., 1991). The alpine ecosystems in the Himalayan region are mostly heterogeneous yet fragile and dynamic, displaying an intricate array of successional phases, from pioneer lichen-moss communities to well-established communities (Rawat, 2007). Abiotic and biotic factors influence the patterns of diversity and distribution of plants along altitudinal gradients (McCain and Grytnes, 2010; Thakur et al., 2022a). It is well known that the knowledge of the floristic composition of plant communities, endemism, and their rarity patterns along the environmental gradient is a prerequisite for understanding the conservation value in the high-altitude mountain region (Sekar et al., 2023; Wani et al., 2023; Rawal et al., 2024; Negi et al., 2024). Alexander von Humboldt was the first to describe the altitudinal gradient of vegetation, which led to the basic understanding of species composition and diversity along elevation gradient (Fischer et al., 2011), and thus conservation implications. Many studies have demonstrated altitudinal gradients provide very effective natural experimental conditions for finding long-term ecological responses to environmental changes. It was generally believed that diversity declined linearly with elevation; however, recent work (Rawal et al., 2018; Umair et al., 2023; Rawal et al., 2023, 2024; Negi et al., 2024) has highlighted that plant diversity often peaks at mid-elevations, which vary among plants and mountain ranges in different climatic conditions. Ecological gradients play a significant role in structuring and shaping plant species richness along altitudinal gradients as reported from various studies from all over the world and the Himalayan region (Vetaas and Grytnes, 2002; Spehn and Körner, 2005; Thakur et al., 2022b; Wani et al., 2022b, 2023; Rawal et al., 2024). However, studies on plant species richness and diversity patterns in cold desert regions of the Indian Trans-Himalaya are limited (Negi et al., 2018a; Singh and Pusalkar, 2020; Saxena and Rao, 2024).

The Indian Trans-Himalaya is one of the fragile landscapes in the Himalayas and is represented by sparse vegetation (Rodgers and Panwar, 1988; Mehta et al., 2020). In India, approximately 98,980 km^2^ area is covered under Trans-Himalaya, popularly known as the cold desert region and distributed over the Ladakh region in Jammu and Kashmir (82,655 km^2^), Lahaul-Spiti and Kinnaur in Himachal Pradesh (15,000 km^2^), and Byans, Dharma, Mana, Nelang, Niti, etc., in Uttarakhand (1,000 km^2^; Srivastava, 2010; Negi et al., 2018a). Few studies have been reported on the vegetation diversity of the cold desert region of the Indian Himalayan Region (IHR) (Naithani, 1995; Negi et al., 2018a), although there is limited information on Trans-Himalayan areas of Uttarakhand, especially in the Nelang Valley. However, this region is known for rare elements of biodiversity and provides ecosystem services to the sustainable livelihoods of 20–30 million people in the region and many more in adjoining areas. The species richness, distribution, and community structure of cold desert regions are important in understanding the ecological dynamics across the world because of their inaccessibility and remoteness (Rawat et al., 2013; Rawal et al., 2018), so conservation and management planning can be developed.

The Nelang Valley is one of the alpine cold desert regions in the Western Himalayas; this region exhibits close affinities with the Tibetan Plateau in terms of both proximity and species composition (Chandola et al., 2008). Rodgers and Panwar (1988) categorized the valley under the Trans-Himalayan Zone. The area was banned from the common public, except for occasional visits by shepherds. It was reported that permission for shepherds led to excessive grazing by their livestock, over-exploitation of trees for fuel wood, and extraction of wild medicinal plants in the region (Chandola et al., 2008; Chandola, 2011). The Uttarakhand State Government has banned the region from tourists for more than half a century; however, now, certain areas of the region are open for tourism activities. Recent studies have now focused on ecological conditions, patterns of species diversity, and the influence of human activities (Fischer et al., 2011; Negi et al., 2018b, 2024, Pathak et al., 2019; Thakur et al., 2021; Rawal et al., 2024). The rapid loss in floristic diversity and changing patterns of vegetation due to these pressures necessitated the qualitative and quantitative assessments of vegetation along the elevation gradient. The study hypothesizes that the opening of the Nelang Valley for tourists may alter the vegetation composition and may also increase the anthropogenic pressure, which has negative impacts on the biodiversity of the region. Therefore, the present is an attempt to i) explore the ecological condition of the region focusing on floristic diversity and species composition, ii) explore the trend of species richness along the elevation gradient, and iii) assess the threat of high-value medicinal plants. The results of the study will be useful for biodiversity management and conservation planning for the region, in particular, and the alpine cold desert in the Himalayas, in general.

## 2 Materials and methods

### 2.1 Study site

**Figure 1:**
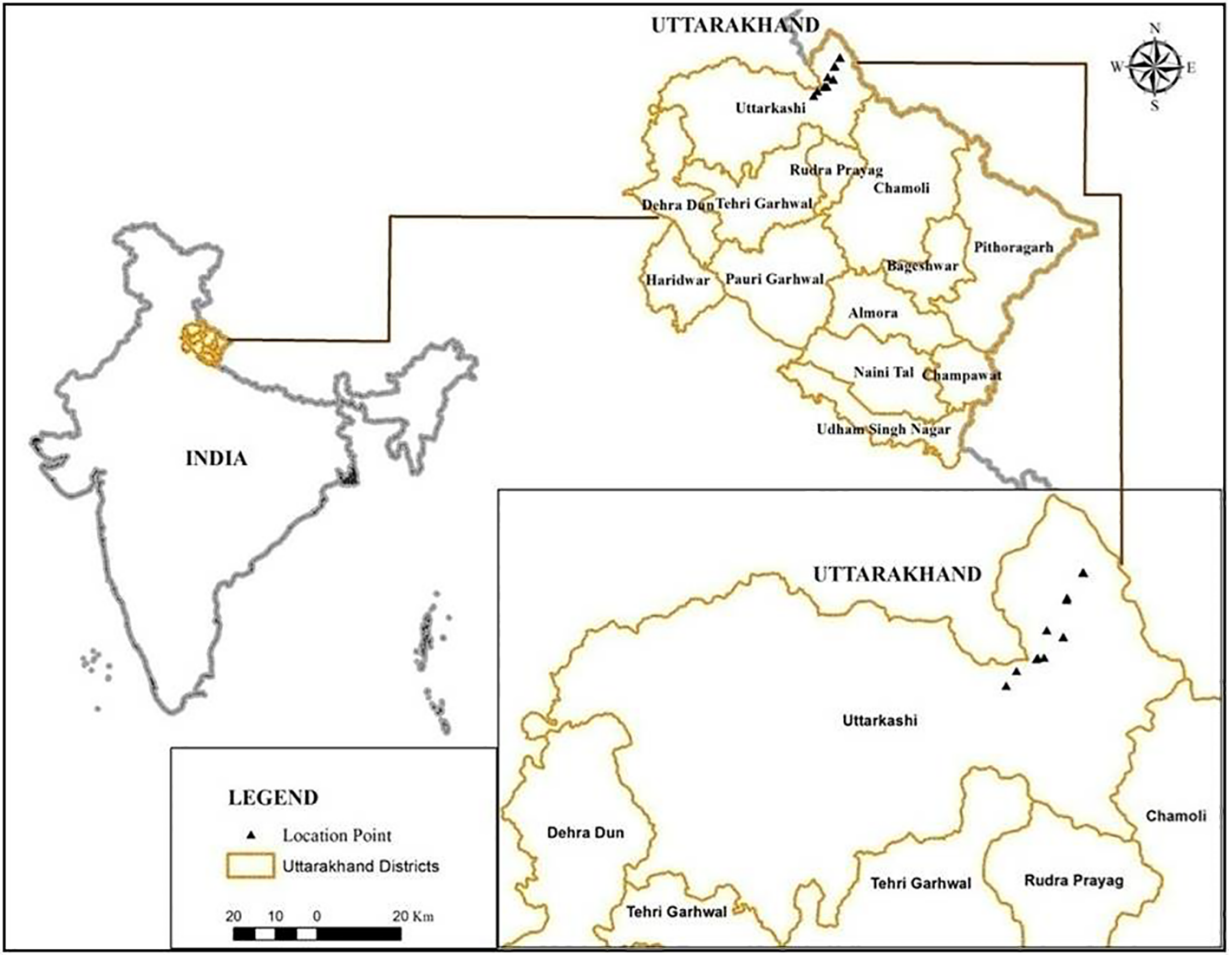
Map of the study area.

The Nelang Valley is a part of the Indian Trans-Himalaya, which lies in the Uttarkashi District of Uttarakhand, India. The valley spreads over an area of approximately 1,360 km^2^, is situated between 31°00′44.1″ to 31°27′06.26″ N latitudes and 78°53′39″ to 79°15′01″ E longitudes, and forms the entire catchment of the river Jadh Ganga (Chandola et al., 2008; **Figure 1**). Given that the valley is a part of the Gangotri National Park and holds an important strategic location, travelers are not allowed to stay overnight. This rule applies within the 25-km area between Bhairavghati and Nelang. In the winter months, the maximum temperature hovers at approximately −5°C to −10°C, while low temperatures are approximately −15°C. Even in the summer months, the maximum temperature does not go beyond 5°C–10°C, and the nightly low temperatures are always in the negative.

### 2.2 Vegetation sampling

A phytosociological analysis of trees, shrubs, and herbs was carried out by stratified random vegetation sampling approach in the valley at every 100-m interval along the elevational gradient (**Table 1**) following Rawal et al. (2018, 2024). The study transects were investigated by systematically dividing transects into 100-m altitudinal belts. Within each 100-m altitudinal belt, forest vegetation was investigated using a systematic random sampling method. In each altitudinal belt, three plots (50 m × 50 m) were marked randomly. For enumerating the vegetation, within each plot (50 m × 50 m), ten 10 m × 10 m quadrats for trees and saplings, twenty 5 m × 5 m quadrats for shrubs and seedlings, and forty 1 m × 1 m quadrats for herbs were laid randomly (Rawal et al., 2018; Negi et al., 2018b; Sekar et al., 2023). In the case of tree species, individuals measuring >10 cm in diameter [diameter at breast height (dbh); 130 cm above ground level] were considered trees (adults), individuals between 3 cm and 10 cm in diameter were considered saplings, and individuals <3 cm in diameter were considered seedlings (Rawal et al., 2018).

**Table 1:** Attributes of studied sites of Nelang Valley.

S. no.	Name of site	Geo-coordinates		Alt. (m asl)	Ni	Ni of different growth forms			SR	G	F	H′	SR of different growth forms		
		Latitude (N)	Longitude (E)			H	S	T					H	S	T
**1**	Site 1	31°03′36.11″	78°56′40.60″	3,100	109	51	21	37	20	19	14	3.92	14	4	2
**2**	Site 2	31°05′31.27″	78°58′12.66″	3,200	76	37	11	28	16	14	11	3.90	10	4	2
**3**	Site 3	31°07′07.00″	79°01′11.44″	3,300	86	56	11	19	20	19	13	3.46	14	5	1
**4**	Site 4	31°07′14.83″	79°01′22.65″	3,400	72	43	7	22	14	14	10	3.88	9	3	2
**5**	Site 5	31°09′59.79″	79°05′05.74″	3,500	71	56	15	0	21	17	10	3.32	14	6	1
**6**	Site 6	31°10′03.26″	79°05′11.15″	3,600	69	56	13	0	21	18	10	3.88	14	6	1
**7**	Site 7	31°10′52.85″	79°02′38.94″	3,700	43	36	7	0	12	11	8	3.81	9	3	0
**8**	Site 8	31°07′18.74″	79°02′20.41″	3,800	59	52	7	0	18	17	11	2.47	14	4	0
**9**	Site 9	31°14′50.71″	79°05′32.75″	3,900	27	18	9	0	14	11	8	3.54	9	5	0
**10**	Site 10	31°15′07.14″	79°05′36.90″	4,000	39	25	14	0	12	11	9	2.82	9	3	0
**11**	Site 11	31°18′25.63″	79°07′57.63″	4,100	26	17	9	0	12	7	5	2.38	9	3	0
**12**	Site 12	31°18′24.26″	79°07′55.17″	4,200	53	53	0	0	7	15	9	1.28	6	1	0
**13**	Site 13	31°18′20.75″	79°08′01.80″	4,300	38	38	0	0	15	13	8	2.52	14	1	0

Alt., altitude; Ni, number of individuals; H, herb; S, shrub; T, tree; SR, species richness; G, number of genera; F, number of families; H′, alpha diversity.

### 2.3 Threat assessment

For threat assessment, a checklist of identified threatened and high-value medicinal plants was prepared. The Rapid Threat Assessment (RTA) was carried out using a rapid vulnerability assessment method employed by Pandey et al. (2019). In the present study, we used a total of five threat criteria to assess each species (i.e., habitat, population size, habitat specificity, life forms, plant parts used, and official threat status). Criterion 1 (habitat of the species) was assessed based on visual observation during the field survey and the available information through literature, and four categories were scored. Habitats like stony and gravel slopes are very fragile; species in these habitats were considered vulnerable to even slight human interference and were hence scored 4 (high threat). Species with less than 250 individuals ha^−1^ and confined to only one locality (elevation level) were considered the most vulnerable (4), while species having more than 1,500 individuals per hectare with occurrence in >5 localities were considered the least vulnerable (1). Based on information collected from local inhabitants/harvesters of medicinal plants and based on available literature, criterion 4 (use pattern) was assessed. In each of the five criteria, scores ranging from 1 to 4 (1 for low and 4 for high threat) were assigned (**Table 2**) per the availability of datasets.

**Table 2:** Vulnerability assessment criteria (Rapid Threat Assessment) for threatened and high-value medicinal plants.

Category of criteria	Sub-category	Scores
Habitat	Gravel/soil, stony/rocky slopes	4
	Moist, marshy, glacial moraine land	3
	Along the stream, agricultural land, shrubberies	2
	Grassland/pastureland/meadows	1
Habitat specificity or occurrence	Rare	4
	Occasional	3
	Sparse	2
	Common	1
Life forms	Long-lived perennial	4
	Short-lived perennial	3
	Biennial	2
	Annual	1
Plant parts used	Bark, stem	3
	Inflorescence, flower, fruit, seed	2
	Leaf	1
Official threat status	Status in 3 categories or more	4
	Status in 2 categories	3
	Status in 1 category	2
	Not assigned	1

### 2.4 Statistical analysis

The quadrat vegetation data were pooled for species richness, density, diversity, and frequency per Rawal et al. (2018). To measure the relative importance of a species in the study area, the important value index (IVI) for the trees was determined as the sum of the relative frequency, relative density, and relative basal area; however, the IVIs of shrubs and herbs were calculated by the sum of the relative frequency and relative density following by Curtis and McIntosh (1950) and Sekar et al. (2023). Relative frequency, relative density, and relative basal area were determined following Rawal et al. (2018). Species richness was determined as the total number of species recorded in sampling plots in each altitudinal interval. The distribution of the plant species in the study area was taken per Chandola (2011). Alpha diversity (H′) was estimated using the Shannon–Weaver index (Shannon and Weaver, 1949) for the establishment of alternative estimates of species diversity in studied sites. Sorenson’s similarity coefficient was calculated following Sorenson (1948) for expressing similarity in plant species composition between different altitudes of the study area. To know the influence of elevational gradient on floristic diversity, distribution, and species composition, polynomial regression was performed using the PAST software (Hammer et al., 2001).

## 3 Results

### 3.1 Floristic diversity and species richness

**Figure 2:**
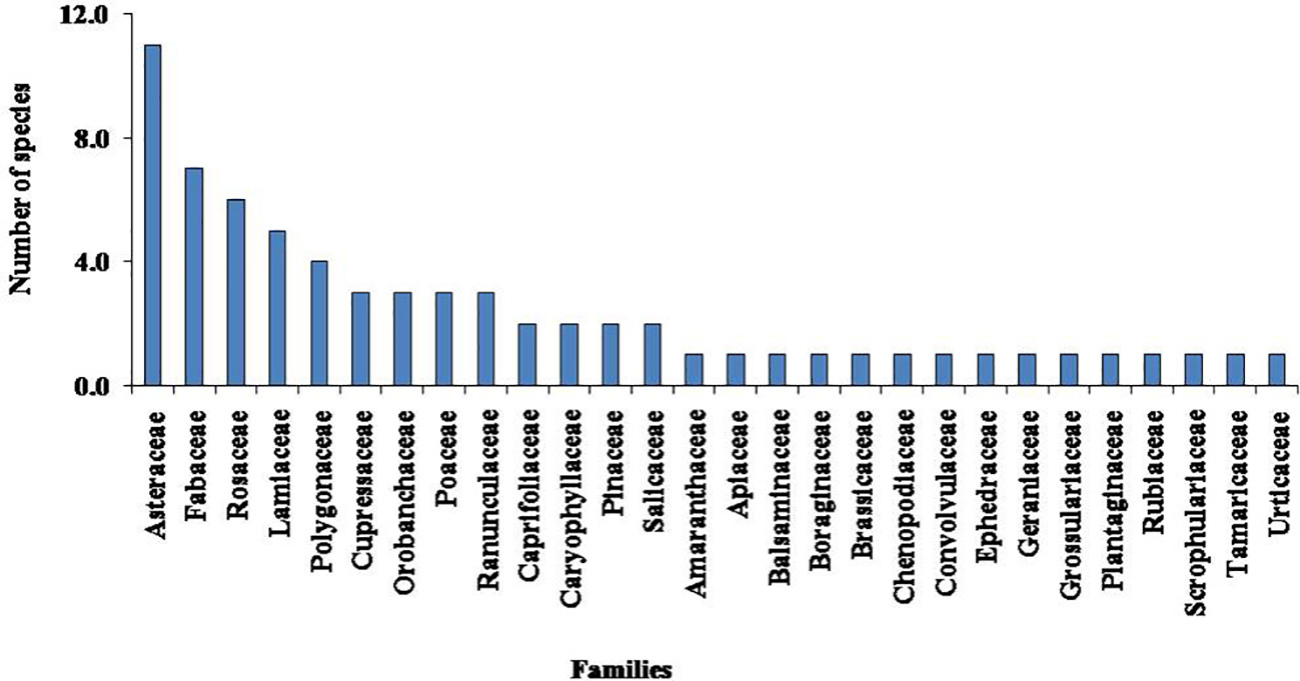
Dominant families with number of species in the study area.

A total of 68 taxa of higher plants were recorded from the study sites in the Nelang Valley (**Table 3**). In addition, species richness of different growth forms such as herbs (51 species), shrubs (13 species), climbers (one species), and trees (three species) were recorded, which represents 56 genera under 28 families (**Table 3**). Artemisia, Astragalus, and Juniperus were the dominant genera, followed by Aster, Lonicera, Oxytropis, Poa, and Salix (**Table 3**). Asteraceae was the dominant family represented by 11 species, followed by Fabaceae (seven), Rosaceae (six), and Lamiaceae (five) (**Table 3**; **Figure 2**). Out of 28 families, 25 were shown with five species or less; however, only three families represent more than five species. Two invasive species, namely, *Convolvulus arvensis* and *Verbascum thapsus*, were recorded from the valley. The herbaceous life forms dominated the entire valley; however, after an altitude of 3,300 m, the trees disappeared or were obscured. In the study area, Juniperus semiglobosa showed dominance with maximum IVI (31.77), followed by Pinus wallichiana (29.19) and Cedrus deodara (10.72; **Table 3**).

**Table 3:** Detailed description of plants that occurred in the study area.

S. no.	Plant name	Family	Life form	Distribution	Flowering and fruiting	Threat status (IUCN)	Important value index (IVI)
1	*Aconogonumtortuosum* (D. Don) H. Hara	Polygonaceae	H	Common	Jun–Sep	–	9.88
2	*Anaphalisnepalensis* (Spreng.) Hand.-Mazz.	Asteraceae	H	Common	Jul–Oct	–	1.65
3	*Aquilegia fragrans* Benth.	Ranunculaceae	H	Sparse	Jul–Sep	–	1.12
4	*Arenaria festucoides* Benth.	Caryophyllaceae	H	Common	Jun–Oct	–	1.59
5	*Artemisia dubia* Wall. *ex* Besser	Asteraceae	H	Occasional	Jun–Aug	–	2.20
6	*Artemisia gmelinii* Weber *ex* Stechm.	Asteraceae	H	Frequent	Jun–Sep	–	5.34
7	*Artemisia roxburghiana* Besser	Asteraceae	H	Occasional	Jun–Oct	–	0.89
8	*Aster albescens* (DC.) Wall. *ex* Hand.-Mazz.	Asteraceae	H	Occasional	Jun–Sep	–	0.79
9	*Aster flaccidus* Bunge	Asteraceae	H	Frequent	Jul–Sep	–	0.31
10	*Astragalus candolleanus* Royle *ex* Benth.	Fabaceae	H	Occasional	Jun–Sep	–	1.25
11	*Astragalus rhizanthus* Royle *ex* Benth.	Fabaceae	H	Sparse	Jun–Sep	–	1.18
12	*Astragalus webbianus* Graham *ex* Benth.	Fabaceae	H	Sparse	Jul–Sep	–	2.07
13	*Bistortaaffinis* (D. Don) Greene	Polygonaceae	H	Common	Jun–Sep	–	0.60
14	*Cedrus deodara* (Roxb. *ex* D. Don) G. Don	Pinaceae	T	Occasional	Sep–Nov	LC	10.72
15	*Ceratoidespapposa* Botsch. & Ikonn.	Chenopodiaceae	H	Occasional	Jun–Aug	–	2.06
16	*Chenopodium botrys* L.	Amaranthaceae	H	Frequent	Aug–Sep	–	2.43
17	*Cicer microphyllum* Royle *ex* Benth.	Fabaceae	H	Common	Jul–Sep	–	7.73
18	*Clematis orientalis* L.	Ranunculaceae	C	Sparse	Jul–Sep	–	0.33
19	*Convolvulus arvensis* L.	Convolvulaceae	H	Frequent	Jul–Sep	–	1.10
20	*Cotoneaster microphyllus* Wall. *ex* Lindl.	Rosaceae	S	Frequent	May–Aug	–	9.51
21	*Cousiniathomsonii* C.B. Clarke	Asteraceae	H	Occasional	Jun–Sep	–	2.56
22	*Cynoglossumglochidiatum* Wall. *ex* Benth.	Boraginaceae	H	Occasional	Jul–Sep	–	0.62
23	*Elsholtziaeriostachya* (Benth.) Benth.	Lamiaceae	H	Frequent	Jul–Sep	–	2.58
24	*Ephedra intermedia* Schrenk *ex* C.A. Mey.	Ephedraceae	S	Common	May–Jun	LC	1.57
25	*Erigeron canadensis* L.	Asteraceae	H	Frequent	Jul–Sep	–	0.39
26	*Eriophytonrhomboideum* (Benth.) Ryding	Lamiaceae	H	Occasional	Jul–Sep	–	2.88
27	*Euphrasia himalayica* Wettst.	Orobanchaceae	H	Frequent	May–Jul	–	4.05
28	*Galium aparine* L.	Rubiaceae	H	Frequent	Jun–Sep	–	0.68
29	*Geranium wallichianum* D. Don *ex* Sweet	Geraniaceae	H	Frequent	Jul–Sep	–	4.61
30	*Hyssopus officinalis* L.	Lamiaceae	H	Rare	Jul–Sep	–	2.57
31	*Impatiens sulcata* Wall.	Balsaminaceae	H	Occasional	Jul–Aug	–	1.15
32	*Juniperus communis* L.	Cupressaceae	S	Common	Sep–Oct	LC	7.38
33	*Juniperus indica* Bertol.	Cupressaceae	S	Common	Jun–Sep	LC	7.16
34	*Juniperus semiglobosa* Regel	Cupressaceae	T	Common	Jun–Sep	LC	31.77
35	*Lonicera asperifolia* Hook. f. & Thomson	Caprifoliaceae	S	Occasional	Jun–Aug	–	1.69
36	*Lonicera spinosa* (Decne.) Jacq. *ex* Walp.	Caprifoliaceae	S	Common	Jun–Aug	–	2.82
37	*Melica persica* Kunth	Poaceae	H	Frequent	Jul–Sep	–	1.62
38	*Myricaria elegans* Royle	Tamaricaceae	S	Occasional	Sep–Nov	–	1.65
39	*Nepeta discolor* Royle *ex* Benth.	Lamiaceae	H	Occasional	Jul–Oct	–	1.75
40	*Orobanche alba* Stephan	Orobanchaceae	H	Occasional	Jul–Sep	–	0.28
41	*Oxyriadigyna* (L.) Hill	Polygonaceae	H	Frequent	Jul–Oct	–	9.79
42	*Oxytropishumifusa* Kar. & Kir.	Fabaceae	H	Occasional	Jul–Sep	–	0.23
43	*Oxytropislapponica* (Wahlenb.) Gay	Fabaceae	H	Occasional	Jun–Sep	–	1.46
44	*Pedicularis punctata* Decne.	Orobanchaceae	H	Occasional	Jul–Oct	–	0.92
45	*Pinus wallichiana* A.B. Jacks.	Pinaceae	T	Common	May–Jul	LC	29.19
46	*Plantago depressa* Willd.	Plantaginaceae	H	Common	May–Jul	–	0.51
47	*Poa alpina*L.	Poaceae	H	Occasional	Jul–Sep	–	1.22
48	*Poa tibetica* Munro *ex* Stapf	Poaceae	H	Occasional	Jul–Sep	–	0.30
49	*Potentilla multifida* L.	Rosaceae	H	Sparse	May–Aug	–	0.69
50	*Ranunculus hirtellus* Royle	Ranunculaceae	H	Common	May–Aug	–	0.59
51	*Ribes alpestre* Wall. *ex* Decne.	Grossulariaceae	S	Occasional	Apr–Sep	–	6.25
52	*Rosa macrophylla* Lindl.	Rosaceae	S	Sparse	Jun–Jul	–	3.29
53	*Rosa sericea* Lindl.	Rosaceae	S	Occasional	May–Aug	–	0.35
54	*Rumex nepalensis* Spreng.	Polygonaceae	H	Common	Jun–Aug	–	5.73
55	*Salix flabellaris* Andersson	Salicaceae	S	Common	Jun–Sep	–	5.58
56	*Salix karelinii* Turcz. *ex* Stschegl.	Salicaceae	H	Occasional	May–Aug	–	7.54
57	*Selinumwallichianum* (DC.) Raizada & H.O. Saxena	Apiaceae	H	Occasional	Jul–Sep	–	2.90
58	*Senecio kunthianus* Wall. *ex* DC.	Asteraceae	H	Occasional	Aug–Oct	–	0.44
59	*Silene moorcroftiana* Wall. *ex* Benth.	Caryophyllaceae	H	Sparse	Jun–Aug	–	0.81
60	*Sisymbrium brassicaeforume* C.A. Mey	Brassicaceae	H	Occasional	Jun–Aug	–	0.59
61	*Sorbaria tomentosa* (Lindl.) Rehder	Rosaceae	S	Common	Jun–Aug	–	1.28
62	*Sorbus aucuparia* L.	Rosaceae	S	Common	Jun–Aug	LC	1.15
63	*Taraxacum officinale* F.H. Wigg.	Asteraceae	H	Sparse	Jun–Sep	–	1.04
64	*Thymus serpyllum* L.	Lamiaceae	H	Common	Apr–Sep	–	3.44
65	*Trigonella emodi* Benth.	Fabaceae	H	Common	Jun–Sep	–	0.56
66	*Urtica hyperborea* Jacq. *ex* Wedd.	Urticaceae	H	Common	May–Aug	–	1.34
67	*Verbascum thapsus* L.	Scrophulariaceae	H	Occasional	Jun–Oct	–	2.77
68	*Youngia glauca* Edgew.	Asteraceae	H	Occasional	Jun–Oct	–	2.09

H, herb; S, shrub; T, tree; C, climber; IUCN, International Union for Conservation of Nature.

Irregular distribution of plant species richness along the altitudinal gradient has been observed. The maximum species were recorded at 3,500 m and 3,600 m asl altitude with 21 species each, followed by 3,100 m asl with 19 species (**Table 1**). The maximum alpha diversity of trees was recorded at 3,000 m (0.64); however, a minimum (0.14) was exhibited at 4,100 m (**Table 4**). Similarly, shrub species showed a maximum (1.66) alpha diversity at 3,500 m, while a minimum (0.33) was recorded at 4,100 m. The mid-elevation (3,800 m) was found best for the alpha diversity (2.58) of the herbaceous layer, and the lowest (0.81) was found at 4,100 m (**Table 4**). Herb, shrub, and tree species show irregular distribution in different altitudes (**Tables 1**, **3**). There is no specific pattern for the diversity of herb species; it was recorded as high at 3,100 m, 3,500 m, 3,600 m, and 3,800 m asl and was minimum at 4,200 m (six species; **Table 1**). In addition, the composition of plants along the altitude was compared using a similarity coefficient based on species richness (**Table 5**). The low-to-high degree of similarity among different altitudes is understandable, as the number of plant species varies from altitude to altitude. Among altitudes, the highest similarity in the composition of plants was recorded between 3,500 m and 3,600 m with the highest number of similar plant species (21 species; 100%; **Table 5**). However, the minimum similarity was shown by 3,400 m and 3,700 m and by 3,400 m and 3,800 with no one similar plant species (0%).

**Table 4:** Species diversity (H') along the altitudinal gradient.

Elevation (masl)	Trees	Shrubs	Herbs	Total
3000	0.64	0.99	2.29	3.92
3100	0.56	1.41	1.93	3.90
3200	0.26	1.09	2.11	3.46
3300	0	1.47	2.41	3.88
3400	0.40	0.96	1.96	3.32
3500	0	1.66	2.22	3.88
3600	0	1.63	2.18	3.81
3700	0	0.67	1.80	2.47
3800	0	0.96	2.58	3.54
3900	0	1.32	1.50	2.82
4000	0	0.35	2.03	2.38
4100	0.14	0.33	0.81	1.28
4200	0	0	2.52	2.52
4300	0	0	2.37	2.37

**Table 5:** Sorenson’s similarity coefficient (in %) among the different altitudinal ranges based on the vegetation.

	3,100	3,200	3,300	3,400	3,500	3,600	3,700	3,800	3,900	4,000	4,100	4,200	4,300
**3,100**	100	59	50	36	40	29	13	5	26	13	14	23	24
**3,200**		100	59	50	16	40	16	6	24	16	17	28	30
**3,300**			100	59	39	39	13	11	26	13	21	29	30
**3,400**				100	34	34	0	0	23	16	17	21	22
**3,500**					100	100	25	32	38	25	47	28	24
**3,600**						100	44	37	31	25	47	32	24
**3,700**							100	43	45	36	30	31	25
**3,800**								100	36	21	8	13	13
**3,900**									100	55	40	31	33
**4,000**										100	30	38	50
**4,100**											100	17	18
**4,200**												100	64
**4,300**													100

### 3.2 Distribution of phytodiversity along the elevation gradient

**Figure 3:**
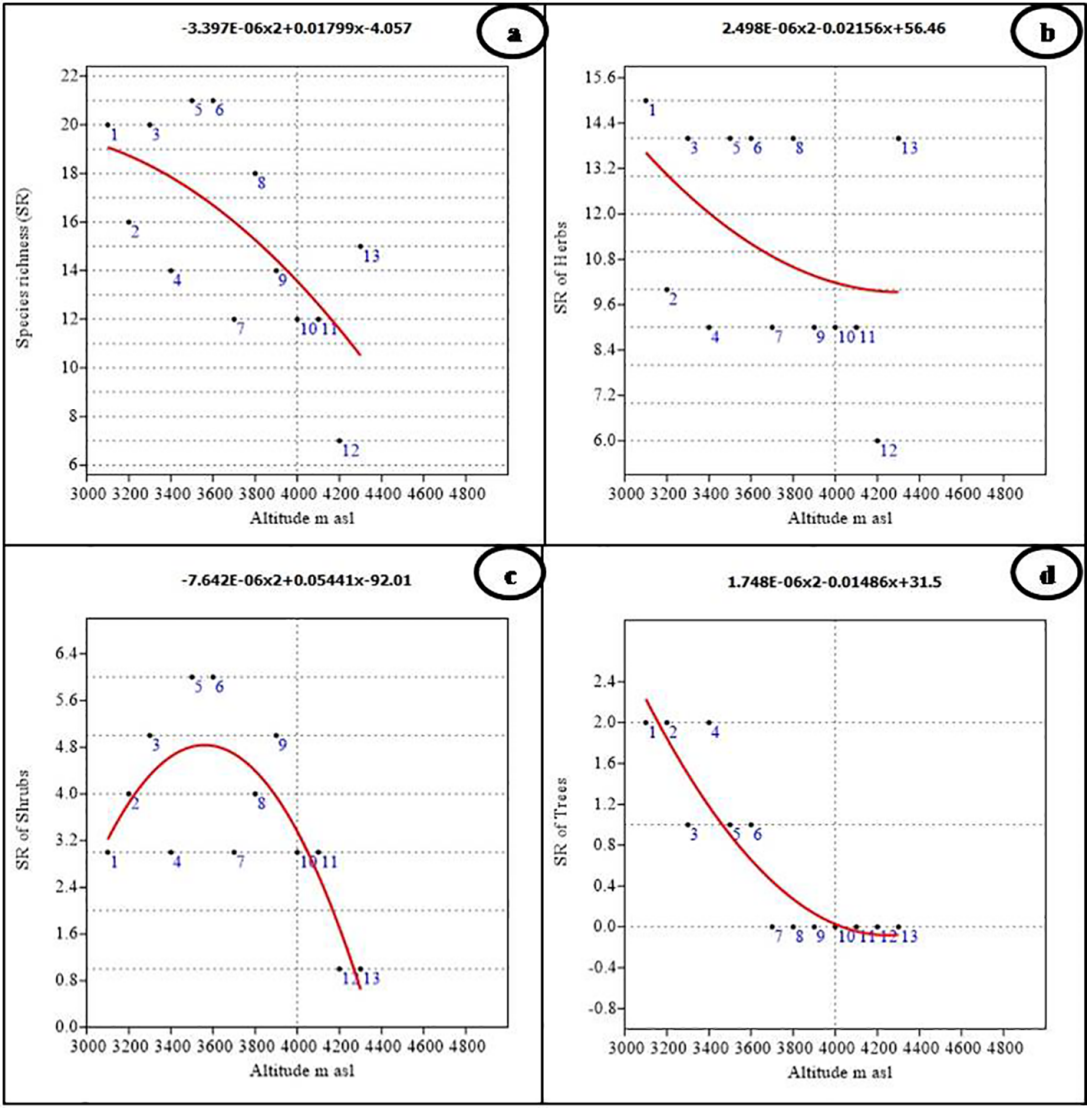
Polynomial regression analysis between altitude and species richness of **(A)** Overall, **(B)** Herbs, **(C)** Shrubs, and **(D)** Trees.

In the Nelang Valley, the total number of phytodiversity individuals exhibited a significant linear decline with increasing altitude. The relationship was monotonic as indicated by the high coefficient of determination (r^2^ = 0.814, p < 0.001, y = 5.375E−05x^2^ − 0.4515x + 986.2). The data, modeled using polynomial functions, showed that the maximum number of individuals (109) occurred at an altitude of 3,100 m. Similar trends were observed for shrubs and trees displaying significant relationships with altitude with a maximum modeled individuals richness of 21 shrub and 37 tree individuals at an altitude of 3,100 m (r^2^ = 0.484, p < 0.05, y = −3.996E−06x^2^ + 0.01935x − 6.794, and r^2^ = 0.912, p < 0.0001, y = 5.245E−05x^2^ − 0.4158x + 821.3, respectively). However, the individual richness of herbs (56 individuals) does not show any relationship with altitude (r^2^ = 0.188, p = 352, y = 5.295E−06x^2^ − 0.055x + 171.7). Species richness across all vegetation types also declined with altitude, showing a significant negative linear relationship (r^2^ = 0.430, p < 0.05; **Figure 3A**). Among the recorded growth forms, herbs were the most taxonomically diverse with 51 species, followed by shrubs (13 species), trees (three species), and climbers (one species; **Table 3**). Shrubs and trees showed significant statistical associations with altitude (r^2^ = 0.645, p < 0.01 for shrubs; r^2^ = 0.837, p < 0.001 for trees), with a clear unimodal, curvilinear relationship observed for shrubs (**Figures 3B, C**). In contrast, trees showed a linear decline in species richness with altitude. Herbs, in contrast, did not exhibit any significant relationship with altitude (r^2^ = 0.173, p = 0.384; **Figure 3D**).

**Figure 4:**
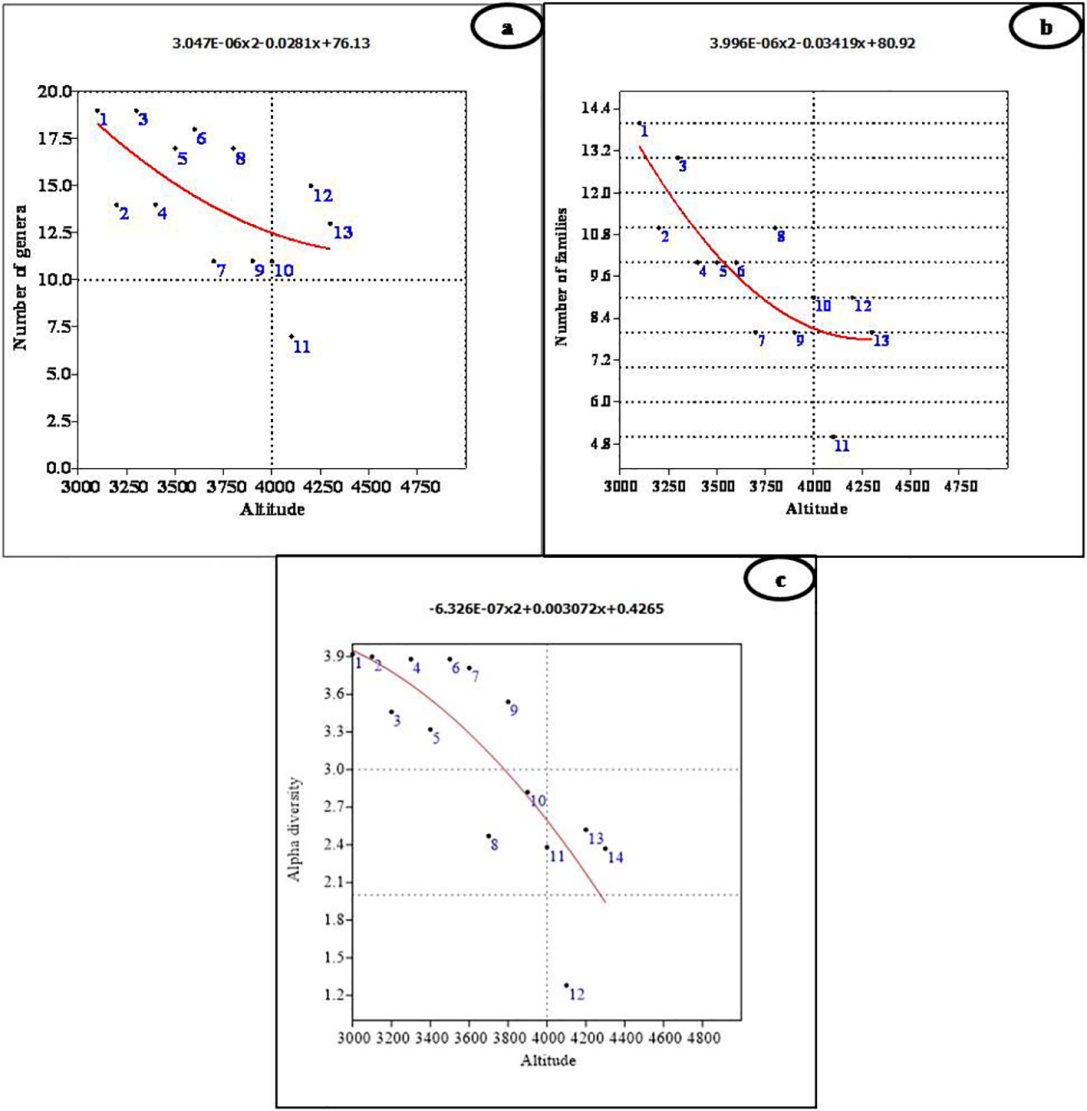
Polynomial regression analysis between altitude and **(A)** number of genera, **(B)** number of families, and **(C)** alpha diversity.

The species richness of different growth forms varied along the altitudinal gradient. Tree species richness was highest at lower altitudes, with a peak of two species between 3,100 m and 3,400 m (**Figures 3B-D**). Beyond 3,600 m, no tree species were recorded, as the alpine meadows began at this elevation. Due to the presence of only one climber species, *viz*., *Clematis orientalis*, it was not possible to assess climber richness along the altitude. The number of genera did not show any significant relationship with altitude (r^2^ = 0.360, p = 0.107; **Figure 4A**), although the highest number of genera (19) was recorded at both 3,100 m and 3,300 m (**Figure 4A**; **Table 1**). However, the number of families demonstrated a significant negative linear relationship with altitude (r^2^ = 0.647, p < 0.01), peaking at 14 families at 3,100 m (**Figure 4B**; **Table 1**). Finally, the Shannon–Weiner diversity index also exhibited a significant negative linear relationship with altitude (r^2^ = 0.647, p < 0.01), with the highest alpha diversity (3.92) observed at 3,100 m (**Figure 4C**; **Table 1**). This suggests that species diversity declines as elevation increases, with the greatest diversity occurring at lower altitudes.

### 3.3 Threat assessment of high-value medicinal plants in the studied alpine region

**Figure 5:**
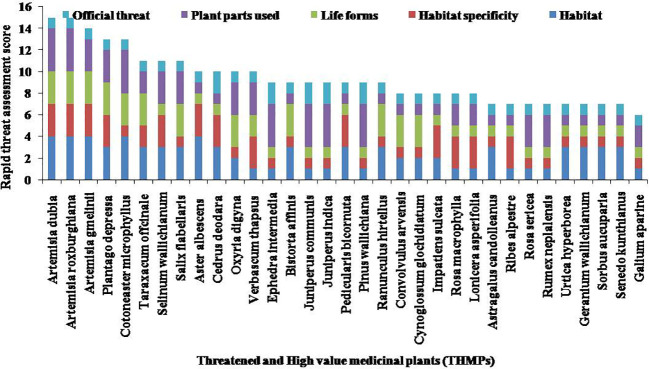
Rapid threat assessment score of THMPs. THMPs, threatened and high-value medicinal plants.

Out of 68 species, 33 species were categorized as medicinally important and traditionally used for curing various types of diseases such as headache, fever, dysentery, diuretics, urinary disorders, and skin diseases by local inhabitants of hilly areas (**Table 6**). Seven species, namely, C. deodara, *Ephedra intermedia*, *Juniperus communis*, *Juniperus indica*, *J. semiglobosa*, *P. wallichiana*, and *Sorbus aucuparia*, were classified as least concern, one of the threatened categories according to the International Union for Conservation of Nature (IUCN 2017). Among the 33 threatened and high-value medicinal plants recorded in the Nelang Valley, *Artemisia dubia* Hara and *Artemisia roxburghiana* Wall. *ex*-Bess. were found most vulnerable with maximum threat score, followed by *Plantago depressa* Willd., *Cotoneaster microphyllus* Wall. *ex* Lindl., and *Taraxacum officinale* F.H. Wigg. (**Figure 5**). These species are included in high-ranking threatened species due to different threats, *viz*., medicinal value, habitat destruction, and other use value patterns. In addition, *Galium aparine* L. scores as the minimum threat and least vulnerable species among the diversity of the Nelang Valley (**Figure 5**).

**Table 6:** Medicinal plants recorded in Nelang Valley.

S. no.	Plant name	Family	Life forms	Used part	Medicinal value	References
1	*Artemisia dubia* Wall. *ex* Besser	Asteraceae	H	Whole plant	Cuts, wounds	Balami, 2004
2	*Artemisia gmelinii* Weber *ex* Stechm.	Asteraceae	H	Whole plant	Headache, boils, pimples	Rokaya et al., 2010
3	*Artemisia roxburghiana* Besser	Asteraceae	H	Whole plant	Eczema, pimples, sores, skin allergy, antipyretic	Gaur, 1999
4	*Aster albescens* (DC.) Wall. *ex* Hand.-Mazz.	Asteraceae	H	Stem	Dysentery	Rawat et al., 2013
5	*Astragalus candolleanus* Royle *ex* Benth.	Fabaceae	H	Root	Blood purifier, cough	Bhatt and Negi, 2006
6	*Bistorta affinis* (D. Don) Greene	Polygonaceae	H	Stem	Dysentery	Rawat et al., 2013
7	*Cedrus deodara* (Roxb. *ex* D. Don) G. Don	Pinaceae	T	Resin, Stem	Rheumatism, ulcer, antihelminthic, astringent, anti-diarrheal and febrifuge, indigestion	Samant et al., 2007
8	*Convolvulus arvensis* L.	Convolvulaceae	H	Root	Purgative	Khan et al., 2013
9	*Cotoneaster microphyllus* Wall. *ex* Lindl.	Rosaceae	S	Root and shoot	Bleeding and PEEP	Khan et al., 2013
10	*Cynoglossumglochidiatum* Wall. *ex* Benth.	Boraginaceae	H	Root	Dyspepsia, digestive disorders	Gaur, 1999
11	*Ephedra intermedia* Schrenk *ex* C.A. Mey.	Ephedraceae	S	Fruit, stem	Fever, cold, flu, and asthma	Gaur, 1999
12	*Galium aparine* L.	Rubiaceae	H	Whole plant	Urinary tract infection	Khan et al., 2013
13	*Geranium wallichianum* D. Don *ex* Sweet	Geraniaceae	H	Root	Astringent, ear and eye diseases, toothache, backache, hepatitis	Qureshi et al., 2009
14	*Impatiens sulcata* Wall.	Balsaminaceae	H	Flower	Pimples	Rawat et al., 2013
15	*Juniperus communis* L.	Cupressaceae	S	Bark, leaf	Kidney complaints, allergy, gas expulsion, and stimulant	Khan et al., 2013
16	*Juniperus indica* Bertol.	Cupressaceae		Leaf, Berries	Fever, cough, and skin ailments	Rokaya et al., 2010; Joshi et al., 2016
17	*Lonicera asperifolia* Hook. f. & Thomson	Caprifoliaceae	S	Fruit	Gastric troubles	Gaur, 1999
18	*Oxyriadigyna* (L.) Hill	Polygonaceae	H	Leaf	Laxative, source of vitamin C	Khan et al., 2013
19	*Pedicularis punctata* Decne.	Orobanchaceae	H	Whole plant	Body ache, sedative, treat flatulence of cattle	Rawat et al., 2013
20	*Pinus wallichiana* A.B. Jacks.	Pinaceae	T	Resin, bark	Abscess, dislocation of joints, ulcers, unconsciousness, diaphoretic, also applied to the cracked (wounded) heels	Khan et al., 2013
21	*Plantago depressa* Willd.	Plantaginaceae	H	Leaf	Dysentery, wounds, and piles	Gaur, 1999
22	*Ranunculus hirtellus* Royle	Ranunculaceae	H	Whole plant	Counterirritant, wound healing	Gaur, 1999
23	*Ribes alpestre* Wall. *ex* Decne.	Grossulariaceae	S	Berries	Heart tonic	Khan et al., 2013
24	*Rosa macrophylla* Lindl.	Rosaceae	S	Flower	Cold and cough, fever, pimples, and skin problems	Negi et al., 2018c
25	*Rosa sericea* Lindl.	Rosaceae	S	Flower	Flower juice is consumed to treat bowel complaints, and salted tea prepared from roots	Negi et al., 2018c
26	*Rumex nepalensis* Spreng.	Polygonaceae	H	Root	Diarrhea and dysentery	Khan et al., 2013
27	*Salix flabellaris* Andersson	Salicaceae	S	Bark	Fever	Rawat et al., 2013
28	*Selinumwallichianum* (DC.) Raizada & H.O. Saxena	Apiaceae	H	Root	Cough, asthma	Gaur, 1999
29	*Senecio kunthianus* Wall. *ex* DC.	Rosaceae	S	Fruit	Cold and cough	Gaur, 1999
30	*Sorbus aucuparia* L.	Rosaceae	S	Leaf	Skin ailments	Gaur, 1999
31	*Taraxacum officinale* F.H. Wigg.	Asteraceae	H	Root	Tonics and blood purifiers, ease urination and other kidney disorders	Khan et al., 2013
32	*Urtica hyperborea* Jacq. *ex* Wedd.	Urticaceae	H	Leaf	Gout, blood purifier, jaundice, skin diseases	Kala, 2006
33	*Verbascum thapsus* L.	Scrophulariaceae	H	Whole plant	For bronchitis and asthma, seed used as narcotic	Gaur, 1999

H, herb; S, shrub; T, tree.

## 4 Discussion

The floristic account of the Indian Trans-Himalaya is known from several publications (Naithani, 1995; Pandey et al., 2019; Negi et al., 2018a, b). However, quantitative information on the patterns of plant species richness and diversity along the altitude gradient is lacking for the region. The present study provides information about the pattern of vegetation composition and species richness along the altitudinal gradient (3,100–4,300 m asl) in the cold desert of the Nelang Valley. It was observed that the number of recorded species in each species was found very low as compared to similar cold desert regions of Uttarakhand. For example, 495 species were reported from Niti Valley of Uttarakhand (Kumar et al., 2016) and 166 species from the Spiti cold desert area of Himachal Pradesh, and 647 species were documented from cold desert Ladakh in Jammu and Kashmir (Kala, 2011). This may be attributed to limited research work from the region due to the ban on human entrance for almost five decades. Naithani (1984) was the first botanist to report 170 species of flowering plants from this valley, followed by Chandola (2011), who reported 446 species. The limitation of our study is that we did not explore the whole valley due to research permission for a few days. Further, our study was focused on the impact of altitude on the species richness and distribution of higher plants. Few researchers documented the alpine vegetation of the Greater Himalayas, and they observed that it is more diverse as compared to the Trans-Himalaya; however, several ethnobotanical studies have been conducted in Garhwal Himalaya except in the Nelang Valley (Chandola, 2011). The topography of the valley, seasonal human pressure in the form of the presence of large camps of ITBP and BRO, and grazing by livestock were reported to have a significant impact on the composition and distribution of vegetation of the area (Chandola et al., 2008; Chandola, 2011).

Asteraceae was the dominant family in the region; other families represent less than seven species. Asteraceae was reported as the most dominant family in the higher elevation range at Spiti (Singh et al., 2007; Sekar and Srivastava, 2009), Himachal Pradesh, and another part of Trans-Himalaya (Kala, 2006; Kumar et al., 2016). Our study also reported Asteraceae as a dominant family in the cold desert region of the Nelang Valley. The dominance of Asteraceae in high altitudes especially in cold desert regions is attributed to the potential of the species to grow in a wide range of environmental conditions (Singh et al., 2007). The irregular distribution of the species in higher altitude zones of the cold deserts was attributed to harsh weather conditions, environmental factors, biotic–abiotic pressure, topography, etc (Singh et al., 2007; Maikhuri et al., 2015; Saxena et al., 2011; Sekar et al., 2014; Negi et al., 2018a, b). The herbaceous layers covered mostly the entire valley, and the presence of a larger number of herbaceous families as compared to shrub species was attributed to their alpine nature, and a similar trend was reported in the Johar and Byans Valleys (Sekar et al., 2014; Negi et al., 2018a, b).

The study finds that altitude also plays a key role in structuring the diversity pattern of vegetation in cold desert regions. The present study reveals the significant impact of altitude on species diversity with a monotonic decline in species richness, and a significant trend was also observed in different regions of the Himalayas (Negi et al., 2018a, c; Rawal et al., 2018, 2024). The maximum species were recorded between 3,500 m and 3,600 m asl in the present study. In mountain areas, such as the Himalayas, the maximum number of endemic species is expected to occur at high elevations due to isolation mechanisms mainly governed by terrains (Acharya et al., 2000; Rawal et al., 2024). The reduction of species in higher altitudes could be attributed to eco-physiological constraints, such as extremely low temperatures, short periods of the growing season, geographical barriers, and lack of adaptability of species to sustain life in hostile climates (Chandola et al., 2008; Pathak et al., 2021). Extreme cold climatic conditions (which remain under snow from November to April) in cold deserts may have influenced the growing season, which further restricted the growth of vegetation (Sekar et al., 2014, 2023). Further, physiological parameters such as the level of moisture and thickness of soil also limit the availability of plant species in cold dry areas (Kala and Mathur, 2002).

Few invasive species are also found in the alpine region of the cold desert of the Nelang Valley, indicating the requirement for conservation measures and management strategies. With increasing tourism and global warming, the spread of invasive species has become a threat to biodiversity (Pathak et al., 2019). It is reported that areas at higher altitudes are more likely to be a refuge for large numbers of species. Given threatened plants, species such as J. semiglobosa are restricted to three locations in the Indian Trans-Himalaya (Sekar et al., 2015) and need proper conservation efforts. Further, the study reported the occurrence of 33 medicinal plants (MPs) from the region, which were mostly extracted from wild populations. Traditional herbal medicine is well recognized in the IHR and plays an important role in healthcare and livelihood enhancement in the rural areas of the region (Dhar et al., 2002; Hamilton, 2004; Kala, 2005; Singh et al., 2021; Negi et al., 2018c, d; Negi et al., 2023). Depletion and loss of MP diversity and associated traditional knowledge base may have significant impacts on human health and livelihood (Wani and Pant, 2023). Hence, protection of the genetic pool of this valuable wealth in nature is urgently required for long-run sustainability, making it available for future generations.

The Nelang Valley is now open for tourists; therefore, it is urgently required to monitor the tourist flow within the carrying capacity of the landscape. Further, sheep and goats are integral parts of summer grazing in alpine areas and have adverse impacts on alpine meadows, especially recruitment of woody taxa in the treeline ecotone. Earlier studies suggested that grazing (Speed et al., 20103), change in grazing pressure (Schickhoff, 2005), and frequent disturbance by tourists may suppress vegetation growth and change their composition (Gottfried et al., 2012). Keeping the importance of the floristic diversity of the Nelang Valley, the following important measures are suggested for the conservation of plant diversity: i) control livestock grazing particularly in treeline ecotone, ii) popularize cultivation of threatened high-value MPs in nearby villages to reduce collection pressure form wild population, iii) promote monitoring of rare and threatened species populations, iv) educate the local inhabitants about the floristic wealth and their conservation value, v) regulate tourist flow according to carrying capacity of the valley, and vi) regularly monitor alpine areas for their management and conservation.

## 5 Conclusions

The Western Himalayan cold deserts are among the most vulnerable zones that are likely to face the worst effects of global warming. However, these regions are habitats of unique and representative biodiversity elements including high-value medicinal plants. The valley shows a high richness of vegetation and a monotonic decline pattern along the altitudinal gradient. The results of the threat assessment highlight the vulnerability of medicinal plants in the studied area, which can be taken into account for prioritizing conservation and management practices. The limitation of our study was that we did not cover the entire valley for vegetation sampling. An exhaustive exploration of the entire valley is therefore required because this valley is in an under-explored condition. The information compiled under the present study has contributed to the vegetation richness and composition of the unique cold desert region of the Western Himalayas, and the information may be used for management and conservation planning.

## Data Availability

The original contributions presented in the study are included in the article/**Supplementary Material**. Further inquiries can be directed to the corresponding authors.
