# Supplementary material for: Floristic diversity and species composition along altitudinal gradient in the alpine ecosystem of the cold desert region in Western Himalaya, India
Source: Front Plant Sci. 2024 Nov 27;15:1469579. doi: 10.3389/fpls.2024.1469579 (PMC11631629; doi:10.3389/fpls.2024.1469579)
Supplement: Supplementary file 1 [file DataSheet1.docx]

**Floristic diversity and species composition along altitudinal gradient in the alpine ecosystem of the Cold Desert Region, in Western Himalaya, India**

Amit Bahukhandi, K. Chandra Sekar*, Vikram S. Negi*, Kapil Bisht, Deep C. Tiwari, Poonam Mehta, Shashi Upadhyay, Sazada Siddiqui, Amel Ayari-Akkari

*** Correspondence:**Dr. K. Chandra Sekar

Email: [kcsekar1312@rediffmail.com](mailto:kcsekar1312@rediffmail.com)

Vikram S. Negi

Email: vikramsnegii@gmail.com

**Supplementary Table 1. Species diversity (H') along the altitudinal gradient**

| Elevation (masl) | Trees | Shrubs | Herbs | Total |
| --- | --- | --- | --- | --- |
| 3000 | 0.64 | 0.99 | 2.29 | 3.92 |
| 3100 | 0.56 | 1.41 | 1.93 | 3.90 |
| 3200 | 0.26 | 1.09 | 2.11 | 3.46 |
| 3300 | 0 | 1.47 | 2.41 | 3.88 |
| 3400 | 0.40 | 0.96 | 1.96 | 3.32 |
| 3500 | 0 | 1.66 | 2.22 | 3.88 |
| 3600 | 0 | 1.63 | 2.18 | 3.81 |
| 3700 | 0 | 0.67 | 1.80 | 2.47 |
| 3800 | 0 | 0.96 | 2.58 | 3.54 |
| 3900 | 0 | 1.32 | 1.50 | 2.82 |
| 4000 | 0 | 0.35 | 2.03 | 2.38 |
| 4100 | 0.14 | 0.33 | 0.81 | 1.28 |
| 4200 | 0 | 0 | 2.52 | 2.52 |
| 4300 | 0 | 0 | 2.37 | 2.37 |


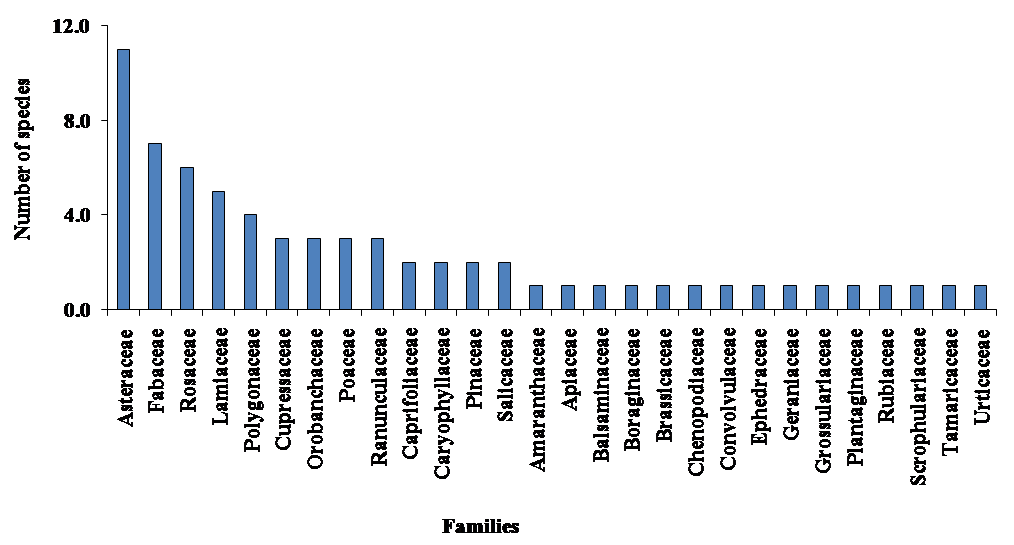


**Supplementary Figure 1**
